# Acute High-Intensity Noise Exposure Induces Cognitive Impairment and Arachidonic Acid Metabolism-Related Molecular Alterations in Rats: A Multi-Omics Study

**DOI:** 10.3390/metabo16020143

**Published:** 2026-02-20

**Authors:** Yane Liu, Mengping Diao, Yihan Hao, Zhongqi Liu, Hao Ma, Yong Zou, Lizhen Ma, Lifeng Wang, Weijia Zhi, Qiong Yu

**Affiliations:** 1 Department of Epidemiology and Biostatistics, School of Public Health, Jilin University, Changchun 130021, China; yeliu23@mails.jlu.edu.cn (Y.L.); diaomp23@mails.jlu.edu.cn (M.D.); yhhao24@mails.jlu.edu.cn (Y.H.); mah24@mails.jlu.edu.cn (H.M.); 2 Beijing Institute of Radiation Medicine, Beijing 100850, China; 20232126112194@stu.usc.edu.cn (Z.L.); zouyong@bmi.ac.cn (Y.Z.); malizhen@bmi.ac.cn (L.M.)

**Keywords:** acute noise exposure, cognitive impairment, multi-omics, arachidonic acid metabolism, oxylipins

## Abstract

**Background**: Acute high-intensity noise exposure represents a critical environmental stressor; however, its impact on brain function and the underlying mechanisms remain incompletely understood. This study aimed to investigate the effects of acute high-intensity noise exposure on cognitive function in rats, utilizing multi-omics analysis to explore potential mechanisms. **Methods**: Rats were exposed to acute noise at 120 dB, and brain function was evaluated using the novel object recognition (NOR) test, recordings of electroencephalographic activity, and histopathological examination. Longitudinal serum metabolomics and fecal metagenomics were performed on samples collected at 0 h, 7, 14, and 28 days post-exposure. Quantitative profiling of oxylipins and proteomics were conducted at a critical time point, followed by integrative multi-omics network analysis. **Results**: Acute high-intensity noise exposure significantly reduced the recognition index in the NOR test, increased theta-band power, and induced hippocampal neuronal damage. Multi-omics analyses revealed time-dependent alterations in gut microbiota and metabolic profiles, identifying day 7 as the critical response window, with arachidonic acid (AA)-derived metabolites consistently downregulated across omics layers. Integrated analysis revealed a coordinated microbiota–oxylipins–proteins network, highlighting key AA-derived oxylipins (e.g., 8-HETE, 12-HETE) that correlated with specific gut microbiota and proteins involved in lipid metabolism and inflammation. **Conclusions**: Acute high-intensity noise exposure induces cognitive impairment and systemic molecular disturbances. AA-centered lipid metabolism acts as a key hub linking gut microbiota dysbiosis with inflammatory and metabolic protein alterations, providing multi-omics evidence for coordinated microbiota–lipid–protein dysregulation underlying noise-induced neurobiological dysfunction.

## 1. Introduction

Noise, recognized as a pervasive environmental pollutant, has emerged as a critical public health concern worldwide. Driven by rapid industrialization and advances in modern military technologies, the risk of acute high-intensity noise exposure has continued to increase. Such exposure is characterized by high sound pressure levels (typically exceeding 100 dB), short duration, and sudden onset, and is frequently encountered in transportation accidents, industrial emergencies, and military operations [1,2,3].

Extensive epidemiological and experimental evidence indicates that noise exposure not only damages the auditory system [4], but also adversely affects the central nervous system by triggering neuroinflammatory responses and oxidative stress [5,6]. These pathological processes contribute to a range of brain functional impairments, including cognitive deficits, anxiety- and depression-like behaviors, and motor dysfunction [7,8,9]. Furthermore, studies have reported that hearing loss is associated with cognitive decline and an increased risk of dementia [10,11]. Accumulating evidence suggests that the brain is a major target organ of noise-induced biological effects [7,12]. For example, previous studies have reported that c-Fos, a marker of neuronal activation, is significantly upregulated in neurons across multiple brain regions—including the cerebral cortex, thalamus, and hippocampus—within two hours of noise exposure, with elevated expression persisting throughout the exposure period [13]. Animal studies further demonstrate that acute high-intensity noise exposure can induce persistent anxiety-like behaviors in both male and female rats [14,15]. Furthermore, Liu et al. [16] reported that a single 2 h exposure to acute noise at 123 dB sound pressure level (SPL) induced long-term cognitive impairment and disrupted hippocampal neurogenesis in mice, accompanied by microglial morphological alterations. Although previous studies have documented the detrimental effects of acute noise exposure on the brain, the molecular pathways linking acute noise to central nervous system dysfunction via gut–brain axis alterations remain largely uncharacterized, and the temporal dynamics of systemic biological responses following acute noise exposure are still poorly understood. Therefore, a systematic, time-resolved characterization of changes in molecular profiles after acute noise exposure is essential for advancing our understanding of its biological effects.

In recent years, advances in high-throughput sequencing and mass spectrometry technologies have enabled multi-omics approaches to emerge as powerful tools for elucidating the biological effects of environmental exposures [17,18,19,20]. Metagenomics and metabolomics have been widely applied to characterize alterations in gut microbiota composition and metabolic profiles under environmental stress conditions [21,22,23,24]. Previous studies have reported that chronic early-life noise exposure alters gut microbial composition and metabolic profiles in rodents, suggesting a potential link between noise exposure and gut-related neural effects [25]. However, whether acute noise exposure in adulthood induces comparable gut-associated molecular responses remains unclear, highlighting the need for further investigation. By integrating multidimensional data from metabolomics, proteomics, and genomics, multi-omics analyses enable high-resolution characterization of exposure-induced molecular alterations. This integrative framework links exposure characteristics with omics responses and health outcomes, facilitating the identification of key injury pathways and potential effect biomarkers. Collectively, such approaches provide robust methodological support for environmental health risk assessment and the development of targeted preventive strategies. Within this study framework, serum and fecal samples were selected to capture systemic metabolic perturbations and gut microbiota alterations associated with acute noise-induced central nervous system dysfunction. These matrices respond rapidly to environmental perturbations, enabling the detection of dynamic molecular changes induced by acute noise exposure. In addition, serum and fecal samples can be obtained through minimally invasive procedures and are readily accessible, providing a practical basis for identifying potential biomarkers of noise-induced injury and offering promising applications in monitoring or early detection. Finally, given the emerging role of the gut–brain axis in mediating peripheral–central communication, integrating serum metabolomics with fecal metagenomics presents a rational strategy for investigating peripheral molecular changes linked to brain functional impairment.

Therefore, in this study, we established a rat model of acute noise exposure to systematically evaluate noise-induced alterations in brain function across multiple levels, including behavioral performance, electroencephalographic activity, and histopathological changes. Concurrently, serum metabolomics, metagenomics, and proteomics were integrated to comprehensively characterize the systemic molecular perturbations induced by acute noise exposure, thereby providing novel insights into the molecular mechanisms underlying noise-related brain dysfunction.

## 2. Materials and Methods

### 2.1. Animals and Experimental Design

A total of 100 eight-week-old male Wistar rats were obtained from SPF Biotechnology Co., Ltd. (Beijing, China). Male rats were used to reduce variability associated with hormonal fluctuations and to better reflect the predominantly male population exposed to acute high-intensity occupational noise. All animals were housed at the Experimental Animal Center of the Military Medical Research Institute under controlled environmental conditions (temperature, 23 ± 2 °C; 12 h light/12 h dark cycle) and were provided with standard laboratory chow and water ad libitum.

**Figure 1:**
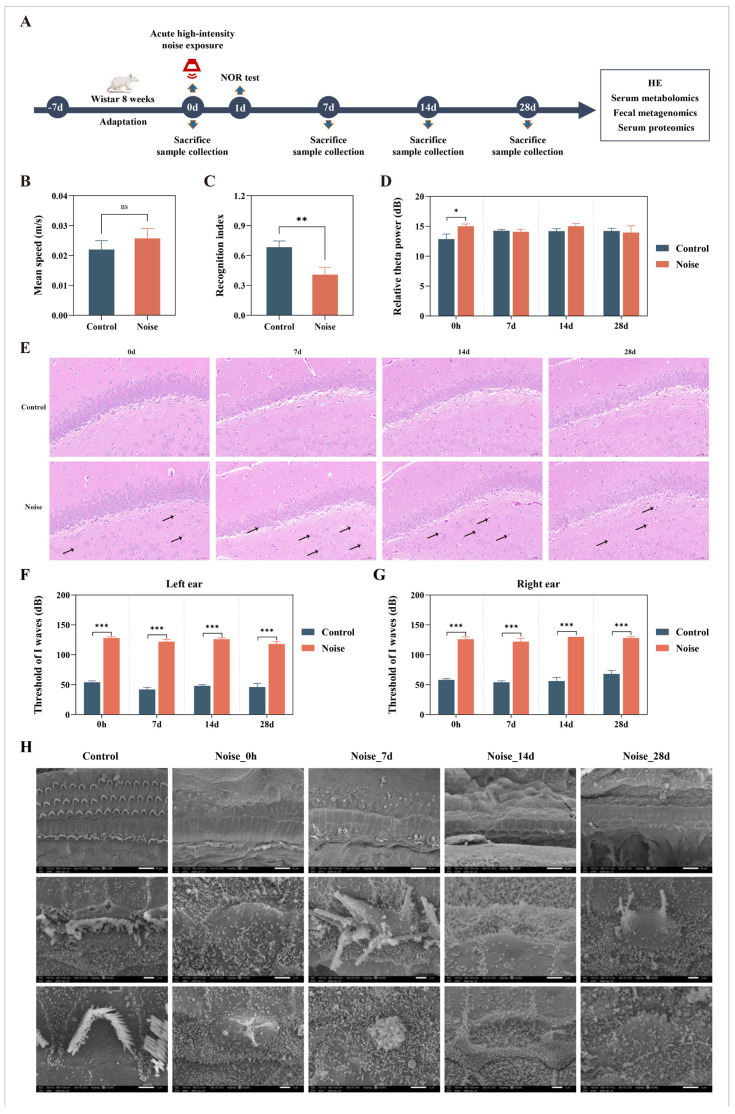
Experimental design and effects of acute high-intensity noise exposure on cognitive and auditory function in rats. (**A**) Schematic overview of the animal experimental design. (**B**) Mean speed and (**C**) recognition index in the novel object recognition (NOR) test after acute high-intensity noise exposure. (**D**) Temporal changes in relative cortical theta band power at 0 h, and 7, 14, and 28 days after noise exposure. (**E**) Representative images of Hematoxylin and eosin (H&E) staining in the rat hippocampus at 0 h, and 7, 14, and 28 days after noise exposure. (Magnification, 40×; scale bar = 50 μm). The arrows in the figure point to neurons with condensed and shrunken nuclei. Impacts of acute high-intensity noise exposure on auditory function in the left (**F**) and right (**G**) ears of rats. (**H**) Representative scanning electron microscopy images of cochlear ultrastructure in rats at the indicated time points following acute noise exposure. Images are shown at magnifications of 1500×, 10,000×, and 15,000×, corresponding to scale bars of 10 μm, 1 μm, and 1 μm, respectively. In the control group, the three rows of outer hair cells were well-organized, with near-oval inner hair cells below. Both inner and outer hair cells exhibited three rows of stereocilia of varying lengths, with the outer hair cell stereocilia arranged in a W-shape. All hair cell stereocilia were intact without breakage, loss, or fusion, and the cuticular plates were complete. In the noise group, both inner and outer hair cells were extensively lost. Data are presented as mean ± sem. * *p* < 0.05; ** *p* < 0.01; *** *p* < 0.001 and ns = not significant.

To comprehensively evaluate the temporal dynamics of acute noise exposure, four post-exposure time points were designated: immediately after exposure (0 h), and 7, 14, and 28 days thereafter. Following a one-week acclimatization period, rats were randomly assigned to either the control group or the noise group (*n* = 50 per group). To avoid potential interference among different experimental procedures, the animals were further allocated to three cohorts for specific assessments: (1) 20 rats for behavioral tests (*n* = 10 per group); (2) 40 rats for electroencephalogram (EEG) recordings (*n* = 5 per group at each time point); and (3) 40 rats for the collection of blood, fecal, and brain tissue samples at the designated time points for histopathological and multi-omics analyses (*n* = 5 per group at each time point). The sample sizes were determined with reference to commonly used group sizes in previous studies investigating the biological effects of acute noise exposure in rodents [14,15,25]. The detailed experimental workflow is illustrated in Figure 1A.

All experimental procedures were approved by the Ethics Committee of the Military Medical Research Institute and were conducted in strict accordance with institutional guidelines for the care and use of laboratory animals.

### 2.2. Noise Exposure

Noise exposure was performed using a noise simulation system developed by the Military Medical Research Institute (Beijing, China). The system consisted of a signal generator, a power amplifier, and an array of loudspeakers mounted on the ceiling of a sound-attenuated chamber. During exposure, rats were housed in custom-designed wire cages positioned directly beneath the loudspeakers. Prior to noise exposure, the sound field was calibrated at the level of the animals’ heads using a sound level meter.

Rats in the noise group were subjected to broadband noise within a frequency range of 40–400 Hz at 120 dB SPL for 4 h, whereas control rats were treated identically except that the noise source remained switched off. Following noise exposure, all rats were immediately returned to their standard housing environment.

### 2.3. Behavioral Experiments

The novel object recognition (NOR) test was used to assess cognitive function in rats and consisted of three phases: habituation, training, and testing. On the day of noise exposure termination, rats were allowed to freely explore an open-field arena (100 × 100 × 40 cm) for 5 min to habituate to the environment. The following day, acquisition training was conducted by placing two identical objects at symmetrical locations approximately 20 cm from the walls of the arena, and each rat was allowed to freely explore for 5 min. One hour after training, a memory recall test was performed, in which one familiar object was replaced with a novel object differing in shape and color, and rats were again allowed to explore the arena for 5 min. To eliminate olfactory cues, the apparatus and objects were thoroughly cleaned with 75% ethanol between trials. Exploration behavior was defined as touching, sniffing, or facing the object within a distance of 2 cm. The time spent exploring the familiar object (T_f_) and the novel object (T_n_) was recorded using VisuTrack behavioral analysis software (V2.0, Shanghai Xinruan Information Technology Co., Ltd, Shanghai, China), and the recognition index (RI) was calculated as RI = T_n_/(T_f_ + T_n_).

### 2.4. Recording and Analysis of Spontaneous EEG

Spontaneous EEG signals were acquired using the MP-160 multichannel physiological recording system (BIOPAC Systems, Inc., Goleta, CA, USA). Rats were anesthetized via intraperitoneal injection of pentobarbital sodium (40 mg/kg) and maintained on a heating pad at 37 °C to preserve body temperature. Needle electrodes were placed at the outer canthi of both eyes and at the midpoint of the interaural line, with a reference electrode positioned on the auricle, and all electrodes were connected to the bioamplifier (BIOPAC Systems, Inc., Goleta, CA, USA). After signal stabilization, EEG activity was recorded for 5 min. The acquired signals were subsequently analyzed using the system’s software and decomposed into four frequency bands: delta (0.5–4 Hz), theta (4–8 Hz), alpha (8–13 Hz), and beta (13–30 Hz).

### 2.5. Recording and Analysis of Auditory Brainstem Response

Following anesthesia under the same conditions as described for EEG recording, auditory brainstem responses (ABRs) were measured using an Intelligent Hearing Smart EP system (Intelligent Hearing Systems, Miami, FL, USA) to assess auditory function. Detailed experimental procedures are described in the Supplementary Materials.

### 2.6. Histological Staining

Brain tissue samples were processed using standard histological procedures, including fixation, dehydration, and paraffin embedding, and were sectioned at a thickness of 3 μm. Hematoxylin and eosin (H&E) staining was used to assess changes in hippocampal tissue.

### 2.7. Preparation of Inner Ear Samples for Scanning Electron Microscopy and Observation of Surface Ultrastructure

Preparation of inner ear samples for scanning electron microscopy (SEM; JEOL Ltd., Tokyo, Japan) was performed using standard procedures. Briefly, temporal bones were harvested at designated post-exposure time points, and cochleae were fixed and processed for SEM analysis. After dehydration and critical point drying, specimens were sputter-coated and examined using a scanning electron microscope to assess cochlear surface ultrastructure. Detailed procedures are provided in the Supplementary Materials.

### 2.8. Serum Untargeted Metabolomics Analysis

Metabolomics, as a phenotype-proximal approach, provides a rapid and sensitive means to capture systemic responses to environmental stimuli and serves as a crucial link between environmental exposure and biological effects [26]. Serum samples collected at the designated post-exposure time points were analyzed using untargeted metabolomics. Detailed procedures are described in the Supplementary Materials.

Differential metabolites (DEMs) were identified by combining univariate *t*-tests with multivariate partial least squares discriminant analysis (PLS-DA). Variables of importance in projection (VIP) derived from PLS-DA, together with statistical significance and fold change (FC) criteria, were used to select DEMs. Specifically, metabolites with VIP ≥ 1, *p* < 0.05, and |log_2_FC| ≥ 0.26 were considered significant. Here, FC was defined as the ratio of the mean relative abundance of each metabolite in the noise-exposed group to that in the control group. Kyoto Encyclopedia of Genes and Genomes (KEGG) pathway enrichment analysis was subsequently performed on the identified DEMs, and pathways with a false discovery rate (*FDR*) < 0.05 were considered significantly enriched.

### 2.9. Fecal Metagenomic Analysis

Fecal samples collected at the designated post-exposure time points were subjected to metagenomic sequencing. Detailed protocols are provided in the Supplementary Materials.

Abundance profiling and differential analyses were performed at the species, functional, and unigene levels. Principal coordinate analysis (PCoA) based on Bray–Curtis distances, combined with PERMANOVA and nonparametric tests, was used to assess differences in overall microbial community structure between groups. Differentially abundant species were identified using MetagenomeSeq (version 1.52.0), with significance defined as |log_2_FC| ≥ 0.58 and *FDR* < 0.05. KEGG pathway enrichment analysis was subsequently conducted for differential unigenes (|log_2_FC| ≥ 1, *p* < 0.05), and pathways with *FDR* < 0.05 were considered significantly enriched.

### 2.10. Quantitative Profiling of Serum Oxylipins

In order to further verify the findings of the non-targeted metabolomics, quantitative profiling of oxylipins was performed on rat serum samples collected on day 7 post-exposure. An LC-MS/MS-based oxylipin analysis platform was used to quantify over 140 oxidized metabolites derived from arachidonic acid (AA), linoleic acid, α-linolenic acid, docosahexaenoic acid, eicosapentaenoic acid, and dihomo-γ-linolenic acid. Detailed experimental procedures are provided in the Supplementary Materials.

Differential oxylipins were identified using criteria consistent with those applied in the untargeted metabolomics analysis, followed by KEGG pathway enrichment analysis.

### 2.11. Serum Proteomics Analysis

Proteomic sequencing was performed using serum samples collected from rats on day 7 post-exposure. Detailed experimental procedures are provided in the Supplementary Materials.

In this study, only proteins detected in at least 60% of samples within each group were retained for subsequent analyses. Missing values were imputed using the k-nearest neighbor (KNN) algorithm. Differentially expressed proteins (DEPs) were identified based on the criteria of |log_2_FC| ≥ 0.58 and *p* < 0.05. Given that proteins exert their biological functions through interactions with other proteins, involvement in signaling pathways, or formation of protein complexes, protein–protein interaction (PPI) networks were constructed using the STRING database to further explore potential molecular regulatory mechanisms. Functional interpretation was integrated with Gene Ontology (GO) and KEGG pathway annotations, with an *FDR* < 0.05 considered statistically significant. PPI networks were visualized and analyzed using Cytoscape software (v3.10.3).

### 2.12. Multi-Omics Analysis

Spearman correlation analysis was performed to assess associations among DEMs, differential gut microbiota, and DEPs. Correlation pairs with an absolute Spearman’s coefficient ≥ 0.6 and *p* < 0.05 were considered significant and used to construct a microbial–metabolite–protein association network, thereby elucidating potential molecular regulatory patterns induced by acute noise exposure.

### 2.13. Statistical Analysis

Data normality was assessed by Kolmogorov–Smirnov test. Based on the characteristics of the data, pairwise comparisons between groups were performed using Student’s *t*-test or Mann–Whitney U test to assess statistical significance. Correlations between variables were evaluated using Spearman correlation analyses. Unless otherwise specified, *p* < 0.05 was considered statistically significant. All statistical analyses and data visualizations were conducted using R software (version 4.5.0) and GraphPad Prism (version 8.0).

## 3. Results

### 3.1. Acute Noise Exposure Induces Cognitive and Auditory Impairments in Rats

Results from the NOR test showed that, compared with the control group, rats in the noise group exhibited no significant differences in speed (Figure 1B) or total exploration time (Figure S1A), whereas the RI was significantly reduced (Figure 1C). These findings indicate that acute high-intensity noise exposure did not affect general locomotor activity but significantly impaired their learning and memory abilities. Dynamic changes in cortical neuronal activity at different post-exposure time points revealed a significant increase in relative theta power immediately following noise exposure (Figure 1D), whereas no significant changes were observed in the delta, alpha, or beta bands (Figure S1B–D). H&E staining further demonstrated that, at multiple post-exposure time points, hippocampal neurons in the noise group exhibited varying degrees of nuclear pyknosis, increased cytoplasmic basophilia, and occasional eosinophilic degeneration (Figure 1E). Furthermore, the number of normal hippocampal cells in the noise groups was significantly reduced compared with the control groups at all post-exposure time points (Figure S1E), confirming the presence of neuronal damage. H&E staining of the auditory cortex also revealed neuronal morphological alterations at different time points following acute noise exposure, characterized by shrunken neuronal somata and condensed, hyperchromatic nuclei in a subset of neurons (Figure S1F). Concurrently, compared with the control group, rats in the noise group exhibited significantly elevated thresholds of I waves at all post-exposure time points (Figure 1F,G), indicating a marked reduction in peripheral auditory sensitivity and definitive hearing loss. SEM provided direct morphological evidence supporting these functional deficits. In control cochleae, the three rows of outer hair cell stereocilia were arranged in a characteristic W-shaped pattern with preserved structural integrity. In contrast, the noise-exposed group displayed progressive structural deterioration. Immediately after exposure and at 7 days post-exposure, cochlear damage was already evident, characterized by widespread loss of outer hair cell stereocilia and fusion of the cuticular plates forming nodular protrusions. At later time points (14 and 28 days post-exposure), cochlear injury further progressed, with complete loss of all three rows of outer hair cells, which were replaced by epithelial cells, accompanied by extensive inner hair cell loss and severe stereociliary degeneration, indicating progressive and irreversible cochlear damage (Figure 1H).

### 3.2. Time-Resolved Serum Metabolomic Profiling Reveals Dynamic Metabolic Responses to Acute Noise Exposure

**Figure 2:**
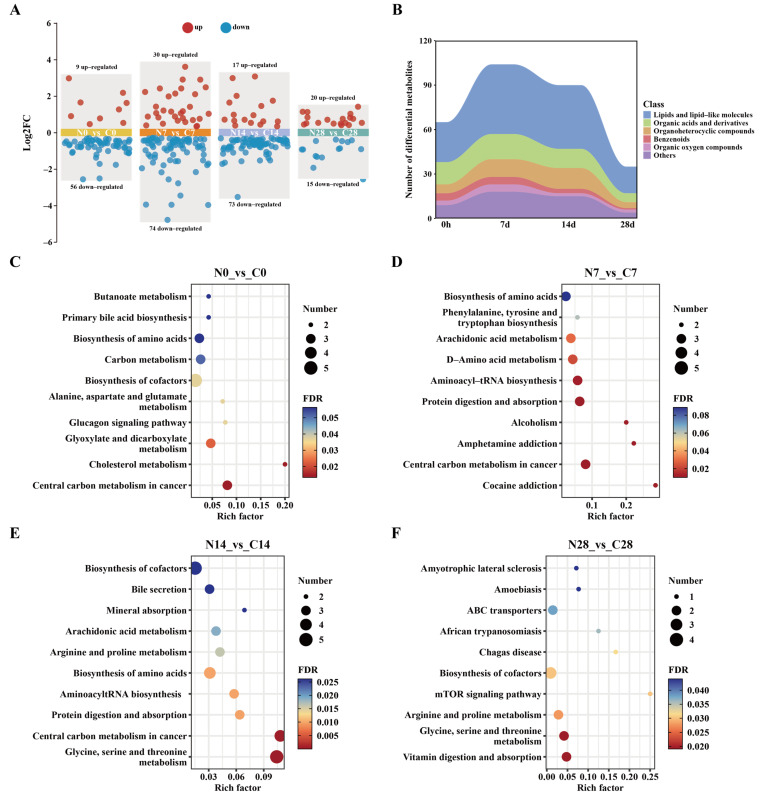
Dynamic metabolic responses to acute high-intensity noise exposure. (**A**) Distribution of differential metabolites (DEMs) in the noise group compared to controls at 0 h, and 7, 14, and 28 days. (**B**) Temporal trends in chemical classifications of DEMs induced by acute high-intensity noise exposure. KEGG pathway enrichment analysis of DEMs at 0 h (**C**), 7 d (**D**), 14 d (**E**), and 28 d (**F**) after noise exposure.

To investigate the potential molecular basis of brain dysfunction induced by acute noise exposure, longitudinal serum metabolomic profiling was performed. DEMs were identified based on strict dual criteria: VIP ≥ 1, *p* < 0.05, and |log_2_FC| ≥ 0.26. Compared with the control group, 65, 104, 90, and 35 DEMs were identified at 0 h, 7 d, 14 d, and 28 d post-exposure, respectively (Figure 2A), with the greatest number observed at day 7. Class analysis of the DEMs revealed that lipid metabolites constituted the predominant category at all time points, accounting for more than 40% of all differential metabolites (Figure 2B). However, intersection analysis indicated a limited overlap of DEMs across different time points (Figure S2).

To further elucidate the biological functions of the DEMs, KEGG pathway enrichment analysis was conducted for each time point. At 0 h, DEMs were primarily enriched in pathways related to energy and lipid metabolism, including glyoxylate and dicarboxylate metabolism, and cholesterol metabolism (Figure 2C). At day 7, DEMs were significantly enriched in multiple pathways associated with neurotransmitter regulation and lipid metabolism, including AA metabolism, aminoacyl-tRNA biosynthesis, and D-amino acid metabolism (Figure 2D). At 14 d, DEMs were mainly enriched in amino acid metabolism and protein synthesis pathways, while also involving lipid metabolism, neurotransmitter signaling (serotonergic synapses), and cellular signal transduction pathways (Figure 2E). Notably, the AA metabolism pathway was significantly enriched at both 7 d and 14 d. At 28 d, DEMs were predominantly enriched in pathways related to amino acid metabolism, signal transduction, and transmembrane transport (Figure 2F). The above results indicate that acute noise exposure induces serum metabolic disturbances in a highly time-dependent manner.

### 3.3. Acute Noise Exposure Induces Time-Resolved Alterations in Gut Microbiota Composition and Function

**Figure 3:**
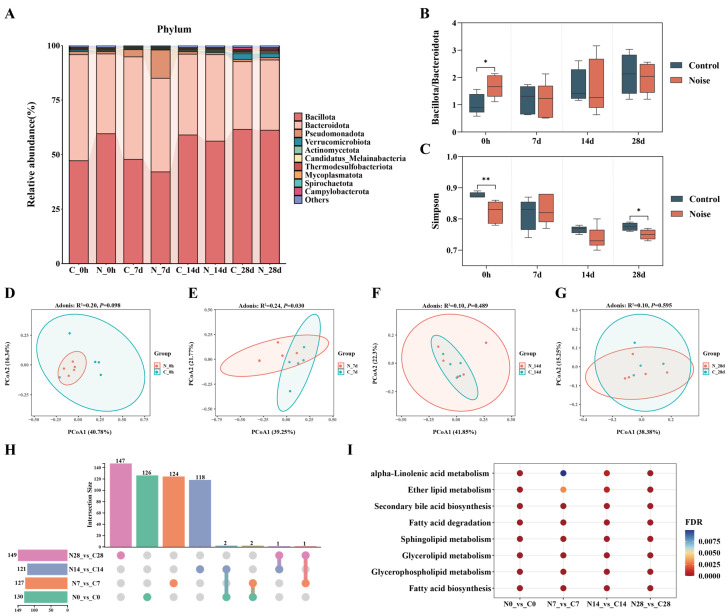
Acute high-intensity noise exposure disrupts the composition and function of the gut microbiota in rats. (**A**) Changes in gut microbiota composition at the phylum level between the noise group and control group at different time points. (**B**) Comparison of the *Bacillota*/*Bacteroidota* ratio between the noise and control groups at different time points after noise exposure. (**C**) Temporal changes in the Simpson index between the noise and control groups. Principal coordinate analysis (PCoA) plot of gut microbiota in the noise and control groups at 0 h (**D**), 7 d (**E**), 14 d (**F**), and 28 d (**G**) after noise exposure. (**H**) UpSet plot of shared and unique differentially abundant microbial species across time points following acute noise exposure. (**I**) KEGG enrichment analysis of lipid metabolism-related pathways consistently altered across all time points. * *p* < 0.05; ** *p* < 0.01.

To investigate the effect of acute noise exposure on gut microbiota, longitudinal metagenomic profiling was performed on fecal samples. As shown in Figure 3A, the top 10 most abundant phyla and their dynamic changes over time revealed that *Bacillota*, *Bacteroidota*, and *Pseudomonadota* were the dominant phyla across all samples. Further analysis showed that at 0 h post-exposure, the *Bacillota/ Bacteroidota* ratio—a classical indicator of gut microbial imbalance—was significantly elevated in the noise group compared with controls (Figure 3B).

Alpha diversity analysis indicated that the Chao1 index did not differ significantly between groups at any time point (Figure S3A), whereas the Simpson index was significantly reduced in the noise group at 0 h and 28 d (Figure 3C), suggesting a decrease in microbial diversity. PCoA analysis revealed a significant separation in microbial community structure between the noise and control groups at 7d (R^2^ = 0.24, *p* = 0.030), whereas no significant differences were observed at other time points (Figure 3D–G). Species-level analysis identified 130, 127, 121, and 149 differentially abundant gut microbes at 0 h, 7 d, 14 d, and 28 d post-exposure, respectively, compared with controls (Figure S3B–E). No differential species were shared across all four time points (Figure 3H), indicating that the impact of acute noise exposure on gut microbial composition exhibits pronounced time-dependent dynamics.

KEGG functional enrichment analysis of differential unigenes further revealed persistent enrichment of multiple lipid metabolism-related pathways across all time points, consistent with the lipid metabolic dysregulation observed in serum metabolomics (Figure 3I). These pathways primarily included fatty acid synthesis and degradation, glycerophospholipid metabolism, glycerolipid metabolism, sphingolipid metabolism, and α-linolenic acid metabolism.

### 3.4. Targeted Oxylipin Profiling Reveals Key Metabolic Alterations at the Day 7 Post-Exposure

**Figure 4:**
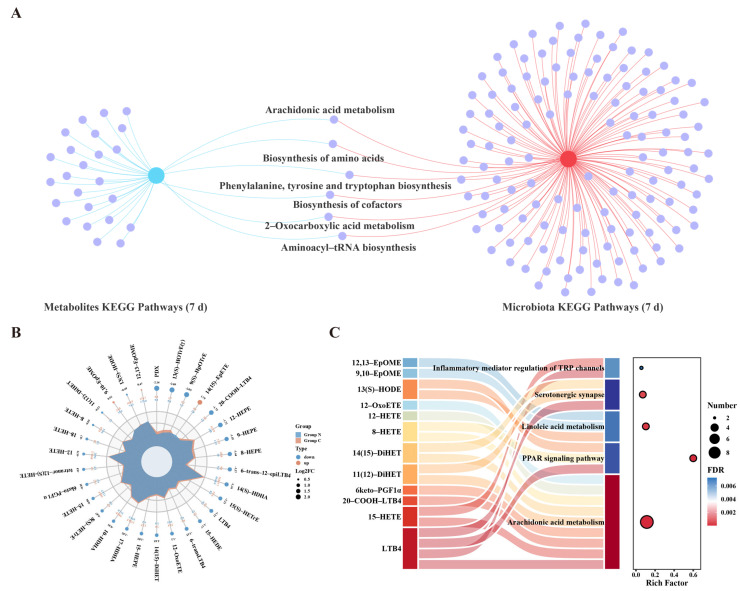
Effects of acute high-intensity noise exposure on serum oxylipin profiles at day 7 post-exposure. (**A**) Venn diagram of significantly enriched pathways identified by metabolomic and metagenomic analyses. Nodes connected to the blue center represent significantly enriched pathways identified in metabolomics, while nodes connected to the red center represent significantly enriched pathways identified in metagenomics. (**B**) Radar chart of differential oxylipins in the noise group (blue area/Group N) compared to controls (orange area/Group C). The dots on the outer circle represent different oxylipins, with orange dots indicating up−regulation and blue dots indicating down−regulation. (**C**) KEGG pathway enrichment analysis of the significantly altered oxylipins.

By integrating the metabolomic and metagenomic findings, we observed that the number of DEMs peaked at 7 d, concomitant with the most pronounced alterations in gut microbial community structure. These results indicate that this time point may represent a critical phase of acute noise-induced molecular responses. Further pathway integration analysis revealed that, at this time point, six pathways were significantly enriched in both metabolomic and metagenomic datasets, among which the AA metabolism pathway exhibited consistent alterations across omics layers (Figure 4A). Based on this cross-omics concordance, we subsequently focused on the critical 7d time point and applied targeted oxylipin profiling to quantitatively validate and characterize the associated lipid alterations.

Compared with the control group, we identified 31 DEMs in the noise group, including 4 upregulated and 27 downregulated metabolites (Figure 4B). Notably, 16-HDHA was undetectable in control samples but was markedly elevated in the noise-exposed group (Figure S4A). KEGG enrichment analysis of the DEMs showed that they were involved in AA metabolism, PPAR signaling pathway, linoleic acid metabolism, serotonergic synapse, and inflammatory mediator regulation of transient receptor potential (TRP) channels (Figure 4C). Among these, the significant enrichment of the AA metabolism further supports its role as a key perturbed pathway underlying acute noise-induced molecular responses.

Furthermore, we found that 12-OxoETE and 12-HEPE were consistently and significantly downregulated in untargeted and targeted metabolomics data (Figure S4B,C). Based on the multi-omics results at day 7, we centered subsequent analyses on the AA metabolism pathway and selected all significantly downregulated metabolites within this pathway identified by targeted analysis—including 12-OxoETE, 20-COOH-LTB4, LTB4, 8-HETE, 15-HETE, 12-HETE, 14(15)-DiHET, 11(12)-DiHET, and 6-keto-PGF1α—as key effector lipid mediators for the construction of a multi-omics interaction network.

### 3.5. Serum Proteomics Reveals Molecular Signatures at the Day 7 Post-Exposure

**Figure 5:**
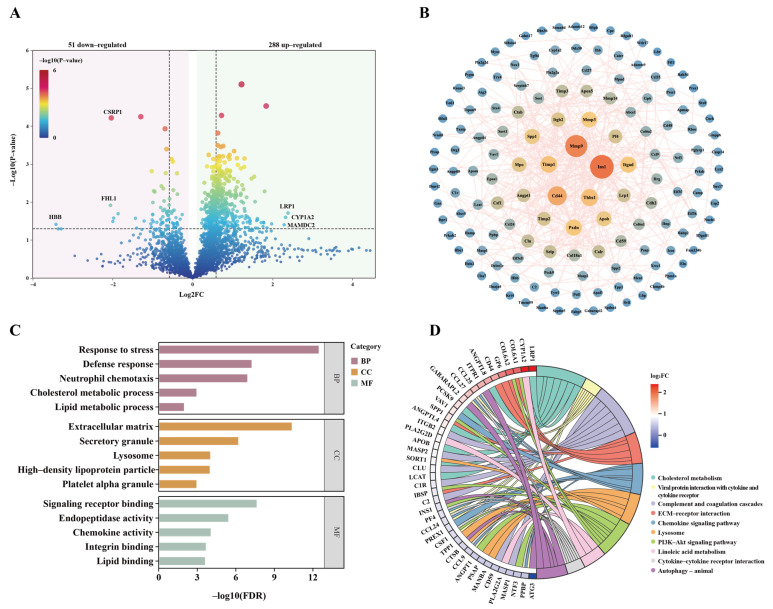
Effects of acute high-intensity noise exposure on serum proteomic profiles at day 7 post-exposure. (**A**) Volcano plot of differentially expressed proteins (DEPs) in the noise group compared to controls. (**B**) Protein–protein interaction (PPI) network of the largest functional module (Cluster 1) derived from network topology-based modular analysis. The nodes represent proteins, and the edges represent interactions. The color and size of the nodes reflect the degree of connectivity; larger, redder nodes indicate higher connectivity (hub proteins), while smaller, bluer nodes indicate lower connectivity. GO (**C**) and KEGG (**D**) pathway enrichment analyses of Cluster 1 proteins.

To further characterize the molecular responses following noise exposure, proteomic analysis was conducted on serum samples collected on day 7 post-exposure. Compared with the control group, a total of 339 DEPs were identified in the noise group, of which 288 were upregulated and 51 were downregulated (Figure 5A). Network topology-based modular analysis categorized these DEPs into 17 functional clusters (Table S1), with Cluster 1 containing the largest number of proteins (*n* = 144). The corresponding PPI network comprised 144 nodes and 423 edges, and PPI enrichment analysis demonstrated significantly higher connectivity than expected by chance (*p* < 1.0 × 10^−16^) (Figure 5B), indicating strong functional associations among the proteins within this cluster.

GO enrichment analysis revealed that Cluster 1 proteins were primarily involved in biological processes related to stress response, immune function, and lipid metabolism (Figure 5C). KEGG pathway analysis further indicated that these proteins were predominantly associated with lipid metabolism and signal transduction pathways, including cholesterol metabolism, linoleic acid metabolism, and the PI3K-Akt signaling pathway, as well as immune-inflammatory and extracellular matrix-related pathways, such as chemokine signaling and complement and coagulation cascades (Figure 5D). Collectively, these findings suggest that acute noise exposure induces systemic alterations in serum proteins, particularly affecting immune-inflammatory responses, lipid metabolic regulation, and remodeling of the cellular microenvironment.

### 3.6. Integrative Multi-Omics Analysis Reveals Coordinated Microbiota–Metabolite–Protein Perturbations Following Acute Noise Exposure

**Figure 6:**
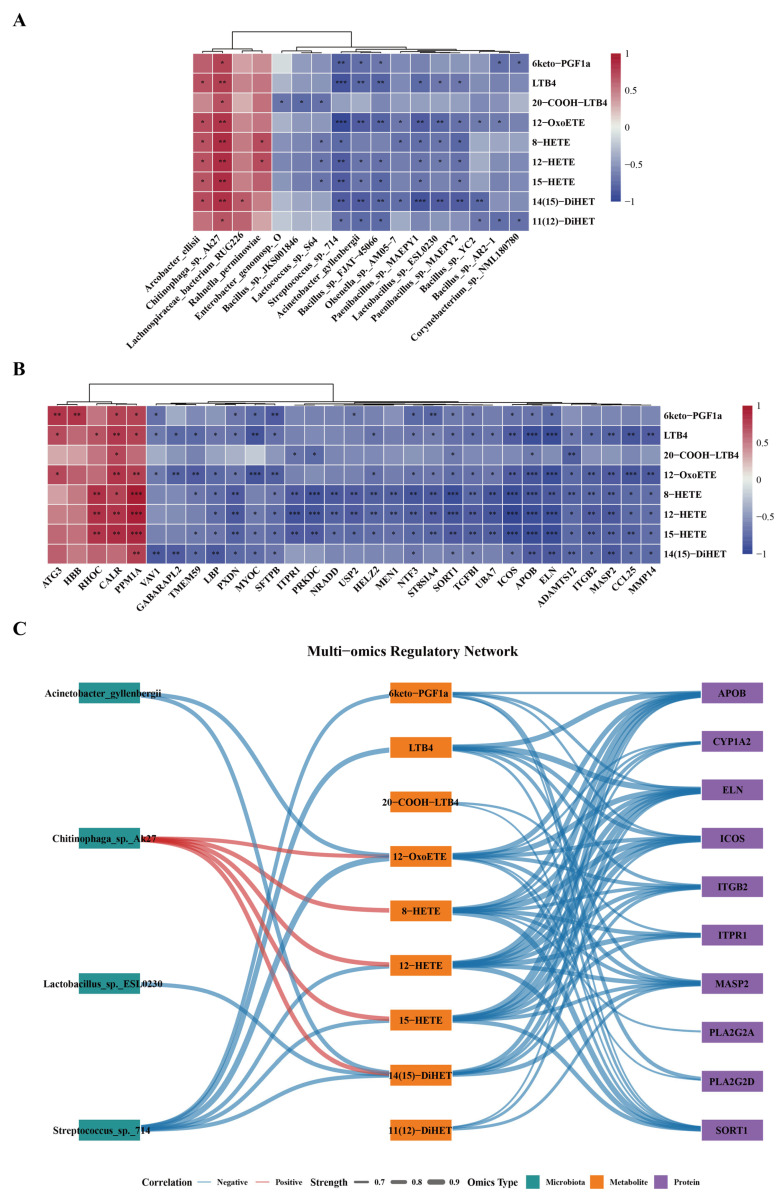
Integrated multi-omics analysis of coordinated microbiota–metabolite–protein perturbations centered on arachidonic acid metabolism. (**A**) Spearman correlation analysis between differentially abundant gut microbiota and key differential oxylipins. (**B**) Spearman correlation analysis between differentially expressed proteins and key differential oxylipins. (**C**) Integrated correlation network of differential gut microbiota, oxylipins, and proteins. * *p* < 0.05; ** *p* < 0.01; *** *p* < 0.001.

Based on the aforementioned omics results, we conducted a cross-omics correlation analysis centered on nine selected metabolites. At the species level, associations between specific gut microbes and key metabolites were first evaluated, with detailed statistical results provided in Table S2. Figure 6A highlights differentially abundant gut microbes exhibiting significant correlations with these metabolites. Concurrently, differentially expressed proteins significantly associated with key metabolites were identified and summarized in Table S3, with strongly correlated pairs (|r| ≥ 0.8) illustrated in Figure 6B.

Integrating correlation strength with molecular functional context, we curated and combined these significant associations, with particular focus on lipid mediators involved in the AA metabolic pathway and their putative functional interactors. This enabled the construction of a microbiota–metabolite–protein interaction network (Figure 6C). In the left panel, key gut microbes significantly associated with critical metabolites are displayed; notably, *Streptococcus* sp. 714 exhibited strong negative correlations with multiple metabolites, including 6-keto-PGF1α, LTB4, 12-OxoETE, 12-HETE, 15-HETE, and 14(15)-DiHET. In the right panel, metabolite–protein associations predominantly showed negative correlations, with lipid metabolism-related proteins (e.g., APOB, CYP1A2, PLA2G2A, PLA2G2D, SORT1) and molecules involved in immune-inflammatory responses and tissue homeostasis (e.g., ICOS, ITGB2, MASP2, ELN, ITPR1) displaying significant inverse correlations with multiple key metabolites.

Overall, LTB4, 12-OxoETE, and HETE metabolites exhibited high network connectivity, serving as hub nodes linking diverse gut microbes and differentially expressed proteins. This integrative network reveals a coordinated perturbation among microbes, metabolites, and proteins, with AA metabolism at its core, under conditions of acute noise exposure.

## 4. Discussion

Acute high-intensity noise is a major environmental factor with detrimental effects on health, yet its impact on brain function and the systemic biological responses it induces remain incompletely understood. In this study, we demonstrate that acute high-intensity noise exposure impairs cognitive function in rats, accompanied by abnormal cortical theta-band activity and persistent structural damage in the hippocampus. Concurrently, acute high-intensity noise exposure induced persistent auditory dysfunction. Multi-omics analyses further reveal that such exposure profoundly disrupts lipid metabolic homeostasis, alters gut microbiota composition and function, and activates immune-inflammatory pathways. Importantly, the multi-omics molecular network constructed in this study uncovers a coordinated perturbation of the gut microbiota, lipid metabolites, and proteins, centered on AA metabolism, providing critical molecular insights for the identification of potential biomarkers associated with noise exposure.

Epidemiological and experimental studies have established that noise exposure adversely impacts neurobehavioral function [5,27,28]. In this study, we demonstrate that a single episode of high-intensity noise exposure impairs cognitive performance in rats. Notably, a previous study using different acute noise conditions (123 dB SPL for 2 h) reported no significant cognitive impairment in mice at early post-exposure stages, with impairments emerging primarily at later stages [16]. In contrast, our results reveal cognitive impairment at an early post-exposure stage. These discrepancies may reflect differences in exposure paradigms, including duration, sound intensity, and spectral characteristics, as well as variations in animal strains or the timing of cognitive assessments. Importantly, they also raise the possibility that acute noise-induced brain functional impairment may depend on a critical exposure duration threshold under specific acoustic conditions. However, a precise time or intensity threshold below which acute noise does not induce measurable neurofunctional damage has not yet been clearly defined. Future studies are needed to systematically determine such exposure thresholds. Additionally, we observed a significant increase in cortical theta-band power immediately following noise exposure. Theta rhythms play a crucial role in attention modulation as well as the encoding of memory, spatial, and temporal information, and their perturbation often reflects disrupted neural network synchrony and altered excitation-inhibition balance [29,30,31]. The hippocampus, a brain region critical for learning and memory and highly sensitive to external stimuli, exhibited histopathological changes across all four post-exposure time points [32,33]. These included neuronal nuclear pyknosis and increased cytoplasmic basophilia, indicating that acute high-intensity noise exposure not only elicits early functional deficits but also causes persistent structural damage, which may underlie the observed cognitive impairments. Additionally, acute noise exposure triggers persistent damage to the auditory system, potentially contributing to cognitive impairment.

Building upon the established detrimental effects of acute high-intensity noise exposure on brain function, elucidating the systemic molecular responses it triggers is of considerable importance. We first examined molecular perturbations from the perspective of serum metabolites. Untargeted metabolomics revealed that acute noise-induced metabolic responses in rats exhibited pronounced time-dependent dynamics, with the number of differentially expressed metabolites initially increasing, peaking at 7d, followed by a decline at 14 d and 28 d. The temporal distribution of DEMs provides insight into the dynamic systemic response to acute noise exposure. Noise exposure can initiate neuroinflammatory cascades and oxidative stress responses that evolve over time rather than occurring instantaneously. Many DEMs were already detectable immediately after exposure (0 h), likely reflecting rapid metabolic perturbations induced by acute stress responses. The peak observed at day 7 may represent an intermediate phase between acute injury and compensatory adaptation, during which sustained inflammatory signaling, membrane lipid remodeling, and disruptions in energy metabolism cumulatively contribute to broader systemic metabolic dysregulation. In contrast, the marked reduction in DEMs at later time points, particularly at day 28, suggests the gradual engagement of compensatory mechanisms and partial restoration of metabolic homeostasis. Notably, lipid metabolites consistently dominated the differential metabolite profiles across all time points, representing a defining feature of acute noise-induced metabolic disruption. Previous studies indicate that lipid dysregulation not only contributes to cardiovascular disease and tumor development but also affects central nervous system function, correlating with deficits in specific cognitive domains such as attention and delayed memory [34,35,36,37]. These findings suggest that alterations in lipid metabolism may serve as a critical mechanistic link between acute high-intensity noise exposure and impaired brain function.

Given the well-established interplay between gut microbiota and lipid metabolism disorder [38], we further investigated microbial responses via longitudinal metagenomic profiling. The composition of the gut microbiota is highly susceptible to environmental influences [39], and such perturbations may subsequently drive gut–brain axis effects. Our study demonstrated that acute noise exposure induced dynamic changes in gut microbial composition and function. Immediately following exposure, the *Bacillota*/*Bacteroidota* ratio was significantly elevated in the noise group. This rapid shift may be associated with acute stress-induced physiological responses. Previous studies have shown that noise exposure can activate the hypothalamic–pituitary–adrenal (HPA) axis and the sympathetic nervous system, leading to elevated corticosterone levels and increased intestinal permeability [40,41,42], thereby potentially contributing to rapid modifications of the gut microenvironment. Notably, as a canonical signature of gut dysbiosis, an increased ratio is not only a well-established feature of metabolic disorders like obesity but also represents a hallmark of microbiota disruption linked to multiple disease risks [43,44]. Furthermore, at 7 d, acute high-intensity noise elicited significant shifts in gut microbial community structure, suggesting this time point may constitute a critical window for noise-induced microbial responses. Functionally, multiple lipid metabolism-related pathways remained consistently perturbed across all time points, closely mirroring the lipid metabolic dysregulation observed in serum untargeted metabolomics.

By integrating temporal and functional patterns from serum metabolomics and gut metagenomics, we identified day 7 as a critical response window. However, structural damage in both the hippocampus and cochlea was observed across all four post-exposure time points. Therefore, the peak observed at day 7 does not indicate maximal structural injury. Rather, this apparent discrepancy reflects the distinct biological layers captured by morphological versus multi-omics analyses. The day 7 molecular peak may represent an intensified phase of molecular dysregulation during the progression of injury. This temporal divergence further supports the notion that molecular perturbations often precede or dynamically accompany structural degeneration. Within this critical response window, AA metabolism emerged as a key pathway mediating acute noise-induced disruption of lipid homeostasis. Targeted oxylipin analysis further confirmed pronounced perturbations in AA metabolism and its associated lipid mediators at this time point. AA metabolism constitutes a central lipid pathway involved in the regulation of inflammation, apoptosis, and signal transduction, and is closely linked to mood regulation, social cognition, and behavior [45,46]. AA is enzymatically metabolized via three major systems—cyclooxygenases (COXs), lipoxygenases (LOXs), and cytochrome P450 (CYP) enzymes—generating a spectrum of bioactive metabolites that modulate inflammatory, immune, and vascular processes [47,48]. Based on this pathway, we identified nine key effector lipid molecules—12-OxoETE, 20-COOH-LTB4, LTB4, 8-HETE, 15-HETE, 12-HETE, 14(15)-DiHET, 11(12)-DiHET, and 6keto-PGF1α—which were consistently downregulated at day 7. These AA-derived metabolites not only act as inflammatory mediators but also serve essential physiological signaling functions. Notably, AA is a polyunsaturated fatty acid critical for central nervous system function. Its oxidative metabolism constitutes a major hallmark of neuroinflammation and has been implicated in cognitive impairment and the pathogenesis of Alzheimer’s disease [49,50,51]. Beyond their roles in AA metabolism, these metabolites participate in key pathways including the PPAR signaling pathway, serotonergic synapses, and TRP channel regulation, all of which are crucial for lipid homeostasis, neuronal signaling, and inflammation regulation. Therefore, the coordinated downregulation of these metabolites may compromise the homeostatic signaling required for synaptic plasticity and neuronal function, thereby contributing to noise-induced cognitive dysfunction.

To further investigate the systemic molecular interactions induced by acute noise exposure, we constructed a cross-omics microbiota–metabolite–protein regulatory network centered on nine key effector lipid molecules. At the microbiota-metabolite level, *Streptococcus* sp. *714* exhibited significant negative correlations with 6-keto-PGF1α, LTB4, and multiple HETEs. Previous studies have shown that *Streptococcus* is highly sensitive to host stress, immune modulation, and environmental perturbations, with abundance shifts often associated with alterations in inflammatory microenvironments or metabolic states [52]. In contrast, *Chitinophaga* sp. *Ak27* displayed significant positive correlations with 12-OxoETE and HETEs metabolites. Members of the *Chitinophaga* genus are recognized as important gut polysaccharide degraders, contributing to host carbohydrate metabolism and intestinal homeostasis [53]. The opposing or concordant associations of these taxa with multiple oxylipins suggest that they may exhibit heightened responsiveness to host lipid metabolic changes under acute noise exposure.

At the metabolite-protein level, several key proteins involved in AA metabolism and its upstream regulation, including CYP1A2, PLA2G2A, and PLA2G2D, exhibited responsive changes. CYP1A2, a critical component of the CYP enzyme system, plays an indispensable role in the metabolism of both endogenous and exogenous compounds [54,55]. Its significant negative correlations with multiple HETEs and 12-OxoETE suggest that acute noise exposure may modulate the dynamic balance of oxylipin signaling through effects on lipid-metabolizing enzymes. Meanwhile, PLA2G2A and PLA2G2D, key upstream enzymes responsible for AA release [56,57], displayed expression changes consistent with a potential disruption of metabolic homeostasis in this pathway. Additionally, several proteins associated with immune and stress regulation, such as ICOS, ITGB2, MASP2, ELN, and ITPR1, were significantly correlated with multiple key lipid mediators. Notably, APOB exhibited strong negative correlations with all nine key lipids. As the primary apolipoprotein of low-density lipoprotein cholesterol, APOB is widely recognized as a marker of cardiovascular disease risk [58]. Emerging evidence further implicates APOB-associated lipid transport dysfunction in cognitive decline and neurodegenerative disease susceptibility, highlighting the critical role of lipid homeostasis in maintaining brain function [59,60,61,62]. Collectively, our findings indicate that acute high-intensity noise exposure not only impairs cognitive performance but also perturbs lipid metabolism and alters APOB-associated lipid and inflammatory proteins, suggesting that dysregulation of lipid homeostasis may constitute a potential molecular basis for noise-induced cognitive impairment.

This study has several limitations. First, our multi-omics analyses were primarily conducted on serum and fecal samples, and the direct relationship between these molecular changes and central nervous system dysfunction induced by acute noise exposure remains to be fully elucidated. Second, due to the use of independent animal cohorts, we were unable to directly correlate individual metabolite levels with cognitive scores. Nevertheless, at the group level, the reduction in AA-derived oxylipins coincided with significant declines in cognitive performance, suggesting a potential functional link. Future studies are needed to further validate the relationship between these lipid alterations and noise-induced cognitive impairment through functional or behavioral correlation analyses. Third, although integrative multi-omics analyses revealed coordinated perturbations among gut microbes, lipid metabolites, and proteins following acute noise exposure, our findings are primarily correlative, and the specific functional roles of key molecules and pathways require further validation through mechanistic experiments. Finally, as this study was conducted exclusively in male rats, the observed findings may not be fully generalizable to females, and future investigations are warranted to explore potential sex-dependent differences.

## 5. Conclusions

In conclusion, our findings demonstrate that acute exposure to 120 dB noise induces cognitive impairment in rats, accompanied by aberrant electroencephalographic activity and hippocampal neuronal injury. At the molecular level, acute noise exposure elicited pronounced systemic perturbations, characterized by alterations in gut microbial community structure, disruption of lipid metabolic homeostasis centered on AA metabolism, and remodeling of the serum proteomic landscape. Notably, the multi-omics association network identifies AA-derived oxylipins as a central hub linking gut dysbiosis with systemic inflammatory protein responses, suggesting that a coordinated peripheral lipid–inflammatory axis may contribute to noise-induced brain dysfunction. Collectively, these findings provide novel molecular insights into the biological basis of noise-induced brain dysfunction.

## Data Availability

The raw data supporting the conclusions of this article will be made available by the authors on request.

## Supplementary Materials

The following supporting information can be downloaded at: https://www.mdpi.com/article/10.3390/metabo16020143/s1. Figure S1: Total exploration time (A) in the novel object recognition (NOR) test after acute high-intensity noise exposure. Temporal changes in relative cortical delta (B), alpha (C), and beta (D) band power at 0 h, and 7, 14, and 28 days after noise exposure. (E) Quantification of normal cells in the hippocampal region of each group at different time points post-exposure. (F) Representative images of Hematoxylin and eosin (H&E) staining in the rat auditory cortex at 0 h, and 7, 14, and 28 days after noise exposure. (Magnification, 40×; scale bar = 50 μm). The arrows in the figure point to neurons with condensed and shrunken nuclei. Figure S2: UpSet plot of shared and unique differential metabolites across time points following acute noise exposure. Figure S3: Effects of acute high-intensity noise exposure on gut microbiota diversity and species composition in rats. (A) Temporal changes in the Chao1 index between the noise and control groups. Distribution of differentially abundant gut microbial species between the noise and control groups at 0 h (B), 7 d (C), 14 d (D), and 28 d (E) post-exposure. Figure S4: Differential metabolites between the noise and control groups. (A) Comparative analysis of the differential oxylipin 16-HDHA in the noise and control groups. Comparative analysis of 12-OxoETE and 12-HEPE levels between noise and control groups as measured by untargeted (B) and targeted metabolomics (C). Table S1: Detailed information on 17 functional clusters derived from network topology-based modular analysis of differentially expressed proteins. Table S2: Statistical results of Spearman correlation analysis between differentially abundant gut microbial species and key metabolites. Table S3: Correlation analysis between differentially expressed proteins and key metabolites.

## Abbreviations

The following abbreviations are used in this manuscript:

NOR	Novel object recognition
AA	Arachidonic acid
SPL	Sound pressure level
EEG	Electroencephalogram
RI	Recognition index
ABR	Auditory brainstem response
H&E	Hematoxylin and eosin
SEM	Scanning electron microscopy
DEMs	Differential metabolites
PLS-DA	Partial least squares discriminant analysis
VIP	Variables of importance in projection
FC	Fold change
KEGG	Kyoto Encyclopedia of Genes and Genomes
FDR	False discovery rate
PCoA	Principal coordinate analysis
KNN	K-nearest neighbor
DEPs	Differentially expressed proteins
PPI	Protein–protein interaction
GO	Gene Ontology
