# Supplementary material for: Acute High-Intensity Noise Exposure Induces Cognitive Impairment and Arachidonic Acid Metabolism-Related Molecular Alterations in Rats: A Multi-Omics Study
Source: Metabolites. 2026 Feb 20;16(2):143. doi: 10.3390/metabo16020143 (PMC12942820; doi:10.3390/metabo16020143)
Supplement: Supplementary file 1 [file metabolites-16-00143-s001.zip › Supplementary Materials.docx]

**Acute high-intensity noise exposure induces cognitive impairment and arachidonic acid metabolism-related molecular alterations in rats: a multi-omics study**

**Recording and analysis of auditory brainstem response**

Subdermal needle electrodes were positioned with the active (recording) electrode inserted behind the tested ear near the mastoid region, the reference electrode placed behind the contralateral ear, and the ground electrode positioned subcutaneously at the midpoint between the two ears. Monaural click stimuli were delivered via an insert earphone at a repetition rate of 19.3 stimuli per second. ABR signals were amplified and band-pass filtered between 100 and 3000 Hz, with 1024 responses averaged per recording. Stimulus intensity was initially set at 120 dB and subsequently decreased in 10 dB steps until wave I was no longer reliably detectable. The lowest intensity at which wave I could be consistently identified was defined as the ABR hearing threshold. Due to the maximum output limitation of the system (120 dB), animals in which no discernible ABR waveform could be elicited at 120 dB were assigned a hearing threshold of 130 dB for subsequent statistical analysis.

**Preparation of inner ear samples for scanning electron microscopy and observation of surface ultrastructure**

At 0 h, 7 d, 14 d, and 28 d after noise exposure, rats were anesthetized with 1% pentobarbital sodium. The temporal bones were rapidly harvested, and the auditory bullae were opened to expose the cochleae. The cochlear specimens were immediately immersed in 2.5% glutaraldehyde fixative and fixed for at least 4 h at 4 °C. After primary fixation, the samples were rinsed with 0.1 M phosphate-buffered saline (PBS) and post-fixed in 1% osmium tetroxide for 2 h at room temperature. The specimens were then rinsed again with 0.1 M PBS and treated with 2% tannic acid for 30 min twice to enhance membrane contrast, followed by an additional rinse in 0.1 M PBS for 1 h. Subsequently, the samples were dehydrated through a graded ethanol series and transitioned with isoamyl acetate. Critical point drying was then performed, followed by ion sputter-coating. Finally, the surface ultrastructure of the inner ear was examined using a scanning electron microscope.

**Serum untargeted metabolomics analysis**

1.Metabolites extraction
Collected samples were thawed on ice, and metabolites were extracted using a 50% methanol buffer. Briefly, 100 μL of each sample was mixed with 400 μL of pre-chilled 50% methanol, vortexed for 1 min, and incubated at −20 °C for 30 min. The mixture was then centrifuged at 420,000 × g for 15 min, after which 400 μL of the supernatant was transferred to a new microcentrifuge tube. Following a second centrifugation at 20,000 × g for 15 min, the resulting supernatant was transferred into autosampler vials for analysis. In addition, 10 μL aliquots from each extract were pooled to generate a mixed quality control (QC) sample. All procedures were performed on ice to minimize metabolite degradation.

2. Liquid chromatography parameters

Liquid chromatography analyses were performed using an ACQUITY UPLC I-Class system (Waters, USA). Metabolite separation was achieved on an ACQUITY UPLC T3 column (100 mm × 2.1 mm, 1.8 μm; Waters, UK). The column temperature was maintained at 40 °C, and the flow rate was set to 0.3 mL/min.

The mobile phase consisted of solvent A, comprising water supplemented with 5 mmol/L ammonium acetate and 5 mmol/L acetic acid, and solvent B, consisting of LC–MS–grade acetonitrile. Gradient elution conditions were set as follows:

| Time | Flow（mL/min） | B％ |
| --- | --- | --- |
| 0.0-0.3 | 0.3 | 5 |
| 0.3-2.0 | 0.3 | 5-70 |
| 2.0-6.2 | 0.3 | 70-99 |
| 6.2-7.5 | 0.3 | 99.0 |
| 7.5-8.0 | 0.3 | 99.0-5 |
| 8.0-10.0 | 0.3 | 5 |

3. Mass spectrometry parameters

High-resolution mass spectrometric data were acquired using a Q Exactive Plus Orbitrap mass spectrometer (Thermo Fisher Scientific, Bremen, Germany). Each sample was analyzed in both positive and negative electrospray ionization (ESI) modes. The ion source was operated with the auxiliary gas (Gas 1) set to 10 and the sheath gas (Gas 2) set to 30, while the sweep gas flow was set to 0. The ion source temperature was maintained at 350 °C. The spray voltage was set to +4.0 kV in positive ion mode and −2.8 kV in negative ion mode. Data acquisition was performed in data-dependent acquisition (DDA) mode. In each acquisition cycle, full MS scans were acquired over an m/z range of 70–1050 at a resolution of 70,000 (at m/z 200), with an automatic gain control (AGC) target of 3 × 10⁶ and a maximum injection time of 100 ms. The top five precursor ions with intensities exceeding 100,000 were subsequently selected for MS/MS fragmentation. MS/MS spectra were acquired at a resolution of 17,500 (at m/z 200), with a maximum injection time of 50 ms. Dynamic exclusion was enabled with an exclusion duration of 6 s. To monitor instrument stability and correct for potential systematic drift, QC samples were analyzed at regular intervals, with one QC injection performed after every ten samples. Mass deviations between QC runs were used to assess and correct batch-level systematic errors.

4. Data preprocessing

The acquired MS data pretreatments, including peak picking, peak grouping, retention time correction, second peak grouping, and annotation of isotopes and adducts, were performed using XCMS software. LC–MS raw data files were converted into mzXML format and subsequently processed using the XCMS, CAMERA, and metaX toolboxes implemented in the R environment. Data quality control and further preprocessing were conducted using the metaX package. Low-quality features were filtered out if they were missing in more than 50% of QC samples or more than 80% of biological samples. The remaining data were normalized using median normalization to reduce systematic variation, followed by imputation of missing values using the minimum value imputation method prior to downstream statistical analyses.

Metabolite annotation was conducted using the online KEGG and HMDB databases by matching the exact molecular mass (m/z) of detected features with database entries. Metabolites were annotated when the mass deviation between the observed value and the database reference was less than 10 ppm, and the corresponding molecular formulas were further confirmed and validated based on isotopic distribution patterns. In addition, an in-house metabolite fragment spectral library was employed to further validate metabolite identification.

The untargeted serum metabolomics analysis was performed with the support of LC Bio Technology Co., Ltd.

**Fecal metagenomic analysis**

Total DNA was extracted from fecal samples using the Fecal Genome DNA Extraction Kit (AU46111-96, BioTeke, China). DNA libraries were constructed with the TruSeq Nano DNA Library Preparation Kit-Set (#FC-121–4001, Illumina, USA) according to the manufacturer’s instructions and subsequently sequenced on an Illumina NovaSeq 6000 platform with PE150 at LC-Bio Technology Co., Ltd. (Hangzhou, China). Sequencing adapters were removed from demultiplexed raw reads using cutadapt (v1.9), and low-quality reads (quality score < 20), short reads (< 100 bp), and reads containing >5% ambiguous bases (“N”) were filtered using the sliding-window algorithm in fqtrim (v0.94). Host-derived reads were removed by aligning quality-filtered reads to the reference genome with Bowtie2 (v2.2). The remaining reads were de novo assembled for each sample using MEGAHIT (v1.2.9). Coding sequences (CDS) were predicted from assembled contigs with MetaGeneMark (v3.26) and clustered across all samples using CD-HIT (v4.6.1) to generate a non-redundant set of unigenes. Protein sequences of the unigenes were aligned against the NR_meta database using DIAMOND (v0.9.14) for taxonomic annotation at multiple classification levels. Functional annotation was performed by aligning unigenes to databases including KEGG and GO. Finally, the abundance profiles of microbial taxa and functional categories were determined based on the unigenes abundance data.

The DNA libraries were sequenced on the illumina NovaseqTM 6000 platform by LC Bio Technology CO.,Ltd (Hangzhou, China). We are grateful to LC Bio Technology CO.,Ltd for assisting in sequencing and bioinformatics analysis.

**Quantitative profiling of serum oxylipins**

1. Chemicals and reagents

All eicosanoids and deuterated internal standards were purchased from Cayman Chemical. HPLC-grade acetonitrile (ACN) and methanol (MeOH) were purchased from Merck (Darmstadt, Germany). MilliQ water (Millipore, Bradford, USA) was used in all experiments. Acetic acid was purchased from Sigma-Aldrich. CNW Poly-Sery MAX SPE cartridges were from ANPEL Co. (Shanghai, PRC). Stock solutions of standards were prepared at a concentration of 5 μg/mL in MeOH and stored at -80°C. Prior to analysis, the stock solutions were diluted with MeOH to obtain working solutions.

2. Sample preparation and extraction

Samples stored at -80°C were thawed on ice. A 200 μL methanol/acetonitrile (1:1, v/v) solution containing internal standards was added to 100 μL of sample and vortexed for 5 min. Proteins were precipitated at -20°C for 30 min. The samples were centrifuged at 12,000 rpm for 10 min at 4°C, and the supernatants were collected. The extraction was repeated once, and the supernatants were combined. Eicosanoids in the supernatants were further extracted using Poly-Sery MAX SPE columns (ANPEL). Prior to analysis, the eluent was dried under vacuum and reconstituted in 100 μL of methanol/water (1:1, v/v) for UPLC/MS/MS analysis.

3. HPLC Conditions

Sample extracts were analyzed using an LC-ESI-MS/MS system (UPLC, ExionLC AD, https://sciex.com.cn; MS, QTRAP® 6500+ System, https://sciex.com). Chromatographic conditions were as follows: column, Waters ACQUITY UPLC HSS T3 C18 (100 mm × 2.1 mm i.d., 1.8 µm); solvent system, water with 0.04% acetic acid (A), acetonitrile with 0.04% acetic acid (B); gradient: 0–2.0 min, 0.1% to 30% B; 2.0–4.0 min, 50% B; 4.0–5.5 min, 99% B, maintained for 1.5 min; 6.0–7.0 min, reduced to 0.1% B and maintained for 3.0 min. Flow rate: 0.4 mL/min; column temperature: 40°C; injection volume: 10 μL.

4. ESI-MS/MS Conditions

Linear ion trap (LIT) and triple quadrupole (QQQ) scans were acquired on a triple quadrupole-linear ion trap mass spectrometer (QTRAP® 6500+ LC-MS/MS System) equipped with an ESI Turbo Ion-Spray interface, operating in negative ion mode, and controlled by Analyst 1.6.3 software (Sciex). ESI source parameters were as follows: ion source, ESI-; source temperature, 550°C; ion spray voltage (IS), -4500 V; curtain gas (CUR), 35 psi. Eicosanoids were analyzed using scheduled multiple reaction monitoring (MRM). Data acquisition was performed using Analyst 1.6.3 software (Sciex), and Multiquant 3.0.3 software (Sciex) was used for quantification of all metabolites. Mass spectrometer parameters, including declustering potentials (DP) and collision energies (CE) for individual MRM transitions, were further optimized. A specific set of MRM transitions was monitored for each elution period according to the metabolites eluted within that period.

Serum oxylipins were detected by MetWare Co., Ltd. using an AB Sciex QTRAP 6500 LC-MS/MS platform.

**Serum proteomics analysis**

1. Enrichment of Low-Abundance Proteins

Low-abundance proteins in each serum or plasma sample were enriched using the magnetic bead method. Briefly, 4 μL of magnetic beads (pre-washed twice with wash buffer) were added to 100 μL of serum or plasma, mixed thoroughly, and incubated for 2 h on a magnetic rack. The supernatant was then removed, and the beads were washed five times with wash buffer for 5 min each.

2. In-Solution Digestion

Proteins bound to the magnetic beads were subjected to in-solution digestion. The beads were lysed in UA lysis buffer (8 M urea, 150 mM Tris-HCl, pH 8.0), followed by reduction with 20 mM dithiothreitol (DTT) at 37 °C for 60 min with gentle shaking. After cooling to room temperature, cysteine residues were alkylated with 50 mM iodoacetamide (IAA) at room temperature for 30 min in the dark. The samples were diluted with 50 mM NH4HCO3 to reduce urea concentration to < 1.5 M. After centrifugation, the supernatant was collected and digested with trypsin at a ratio of 1:50 (w/w) for 16 h at 37 °C. Peptides were desalted using C18 cartridges (Empore™ SPE Cartridges, Sigma), concentrated by vacuum centrifugation, and reconstituted in 20 μL of 0.1% (v/v) formic acid. Peptide concentration was estimated by UV absorbance at 280 nm. For DIA experiments, indexed retention time (iRT) calibration peptides were spiked into each sample.
3. LC-MS/MS Acquisition for Data-Independent Acquisition (DIA)

All data were acquired using a Thermo Scientific Vanquish Neo UHPLC system coupled to an Orbitrap™ Astral™ mass spectrometer equipped with a Thermo Scientific Easy-Spray source, operated in data-independent acquisition (DIA) mode. Chromatographic separation was performed on a 1.9 μm × 150 μm × 15 cm column (ES906). Mobile phase A consisted of 0.1% formic acid in water, and mobile phase B consisted of 80% acetonitrile with 0.1% formic acid in water. Precursor ions were scanned over a mass range of 380–980 m/z. MS1 spectra were acquired in the Orbitrap at a resolution of 240,000 (at m/z 200), with a normalized AGC target of 500% and a maximum injection time of 5 ms. MS2 spectra were acquired in DIA mode using 299 isolation windows, each 2 m/z wide, with HCD collision energy set at 25 eV, normalized AGC target of 500%, and maximum injection time of 3 ms.
4. Mass Spectrometry Data Analysis

DIA data were analyzed using Spectronaut Pulsar 18. Enzyme specificity was set to trypsin, with a maximum of two missed cleavages. Carbamidomethylation of cysteine was set as a fixed modification, while oxidation of methionine and protein N-terminal acetylation were set as dynamic modifications. Proteins were identified with 99% confidence, and the FDR was controlled at ≤ 1%.

The serum proteomics analysis was performed with the support of Applied Protein Technology Co., Ltd.
