# Supplementary figures and images for: Acute High-Intensity Noise Exposure Induces Cognitive Impairment and Arachidonic Acid Metabolism-Related Molecular Alterations in Rats: A Multi-Omics Study
Source: Metabolites. 2026 Feb 20;16(2):143. doi: 10.3390/metabo16020143 (PMC12942820; doi:10.3390/metabo16020143)

**A**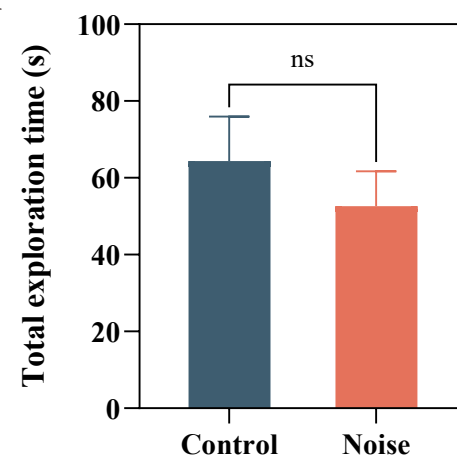**B**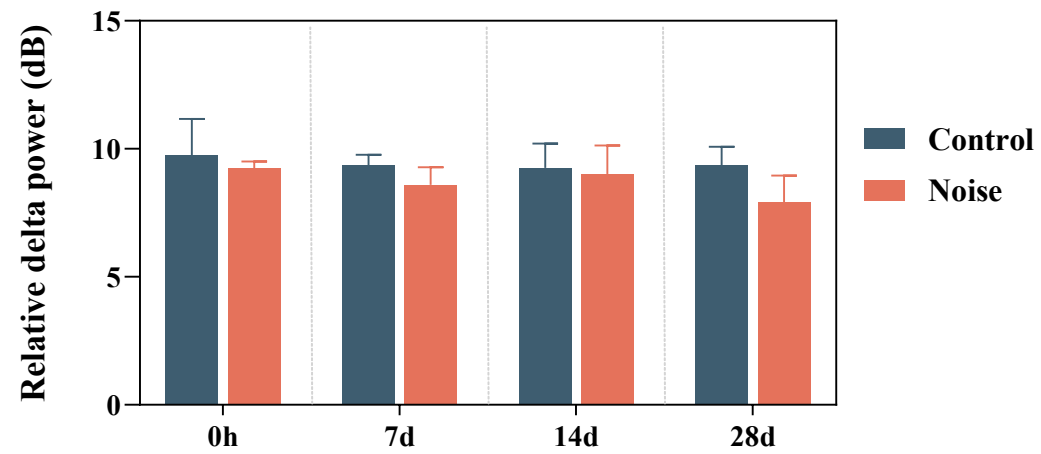**C**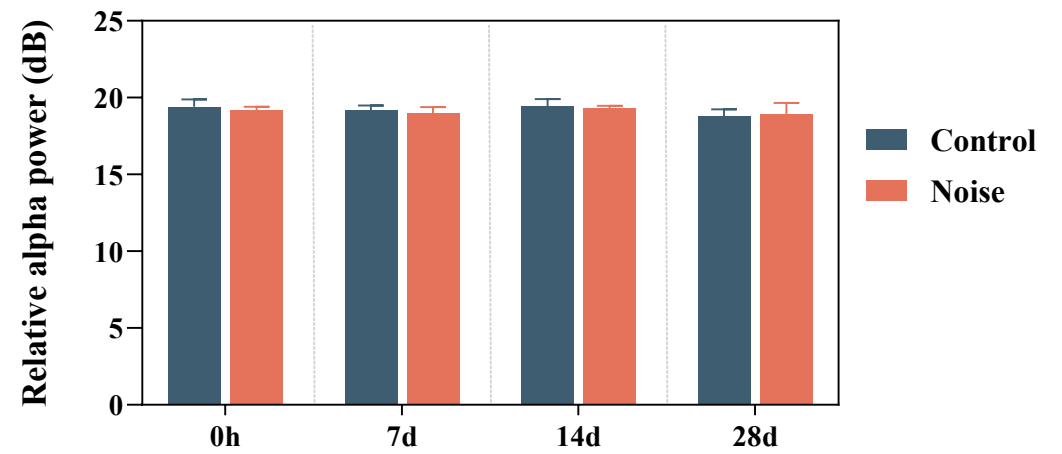**D**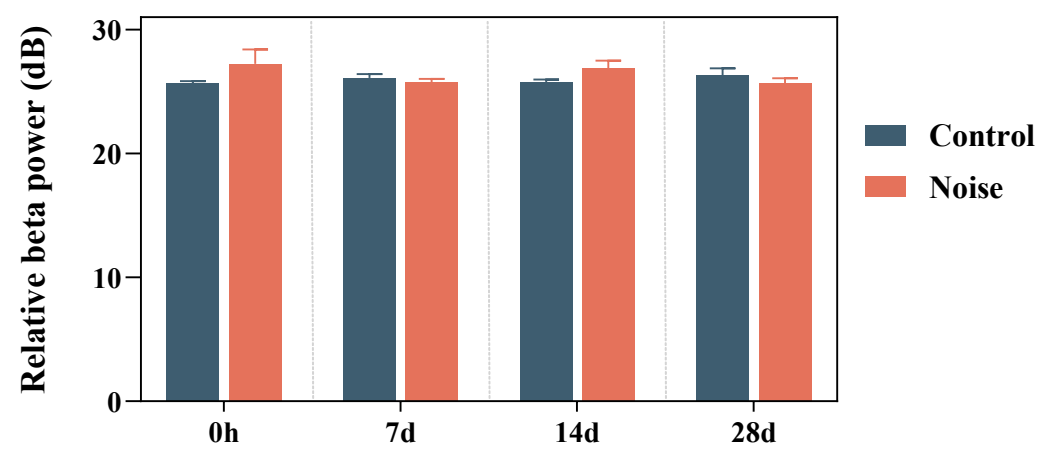**E**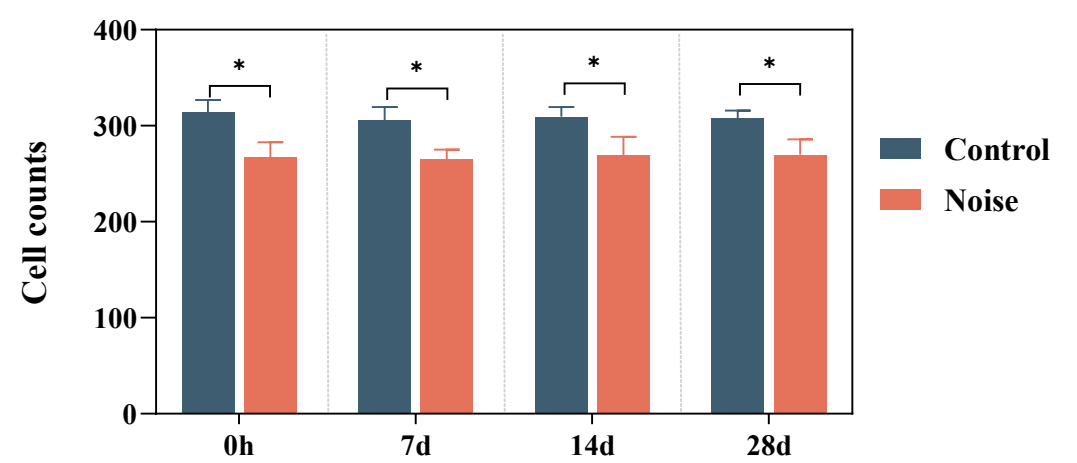**F**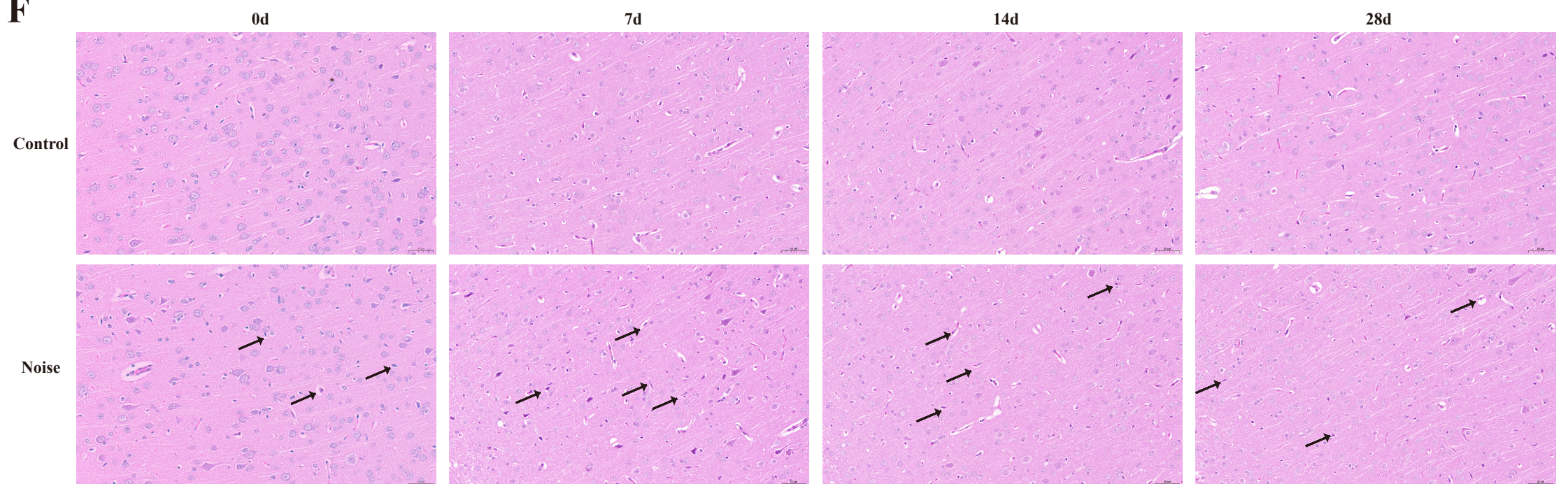

Supplement: Supplementary file 1 [file metabolites-16-00143-s001.zip › Figure S1.pdf]

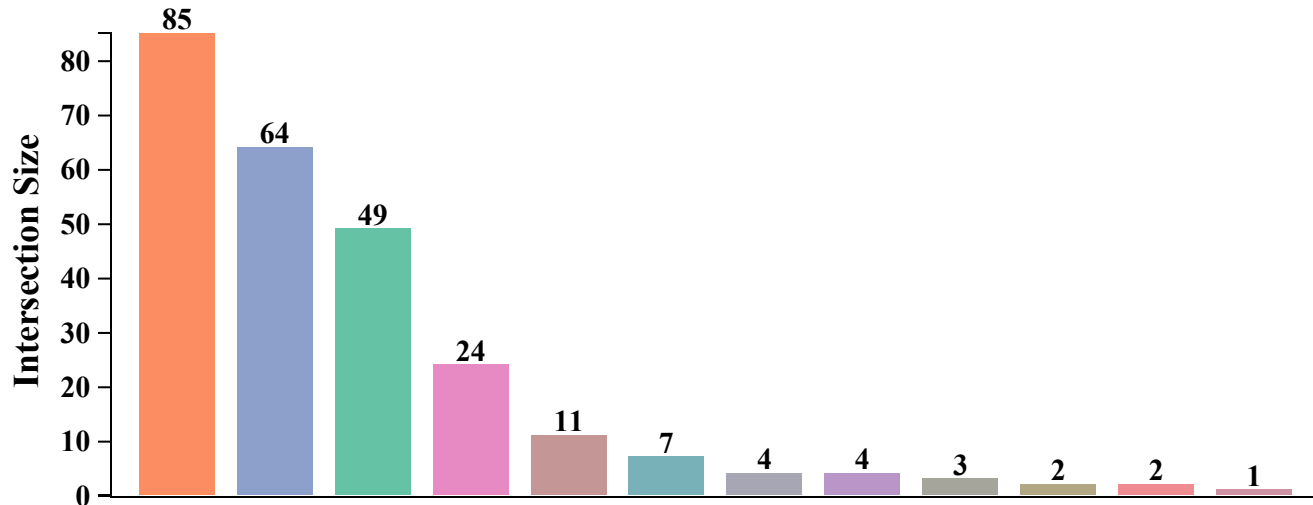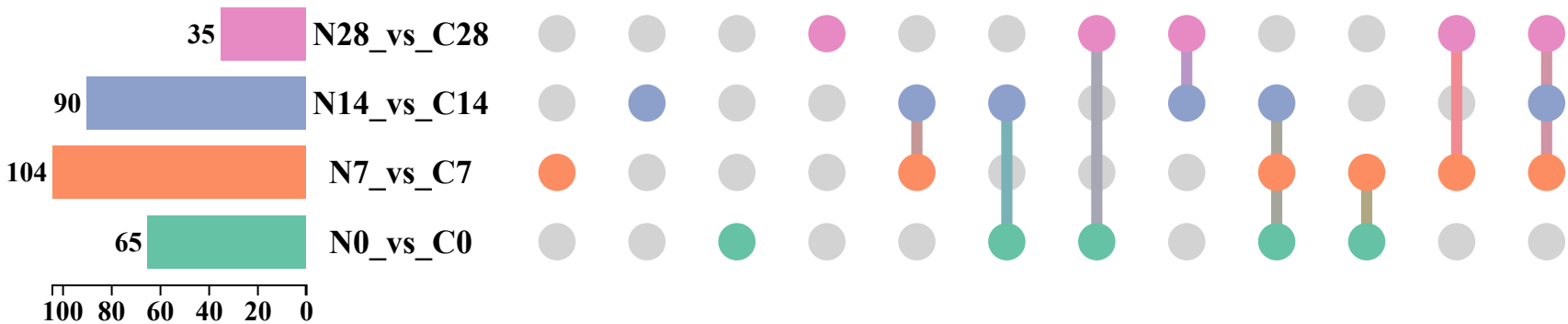

Supplement: Supplementary file 1 [file metabolites-16-00143-s001.zip › Figure S2.pdf]

A

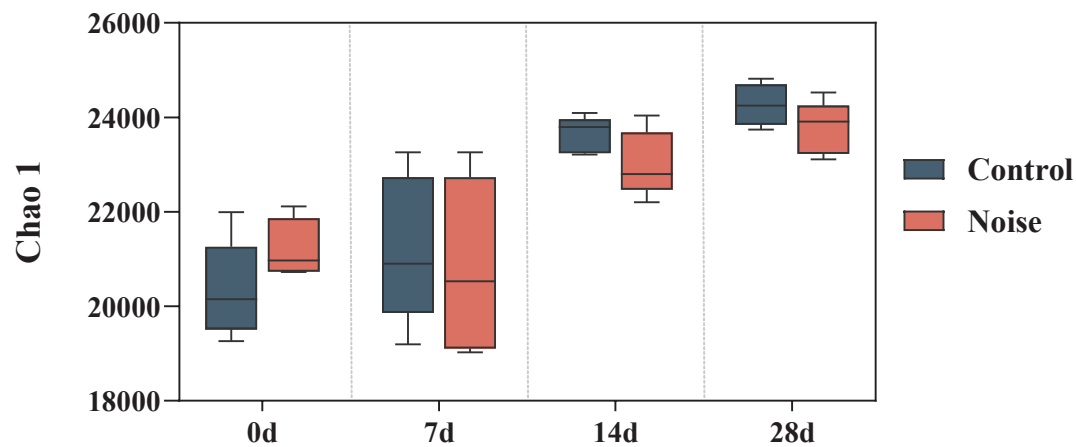

B

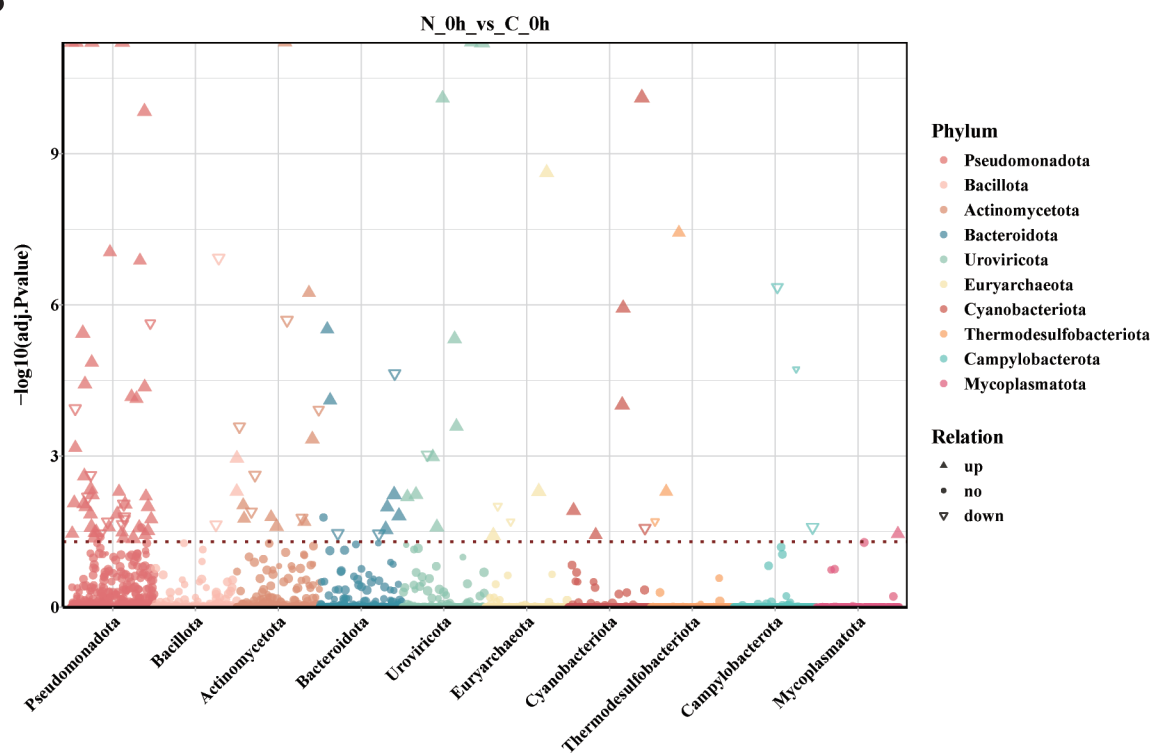

C

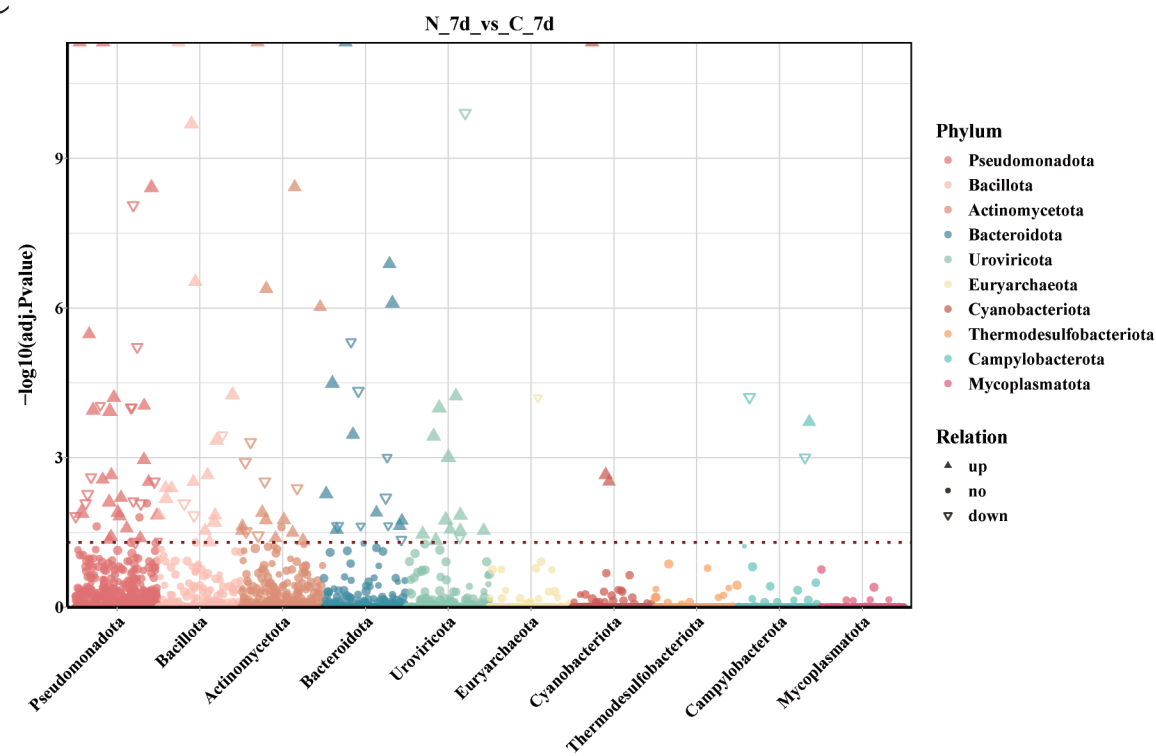

D

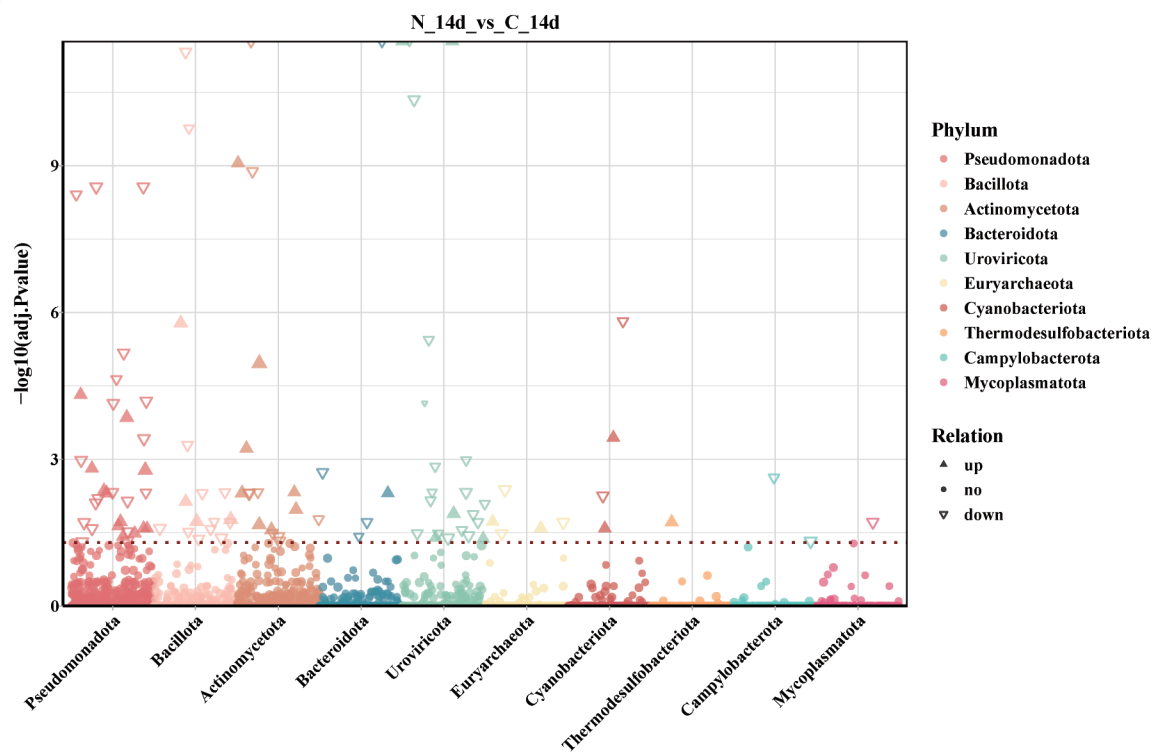

E

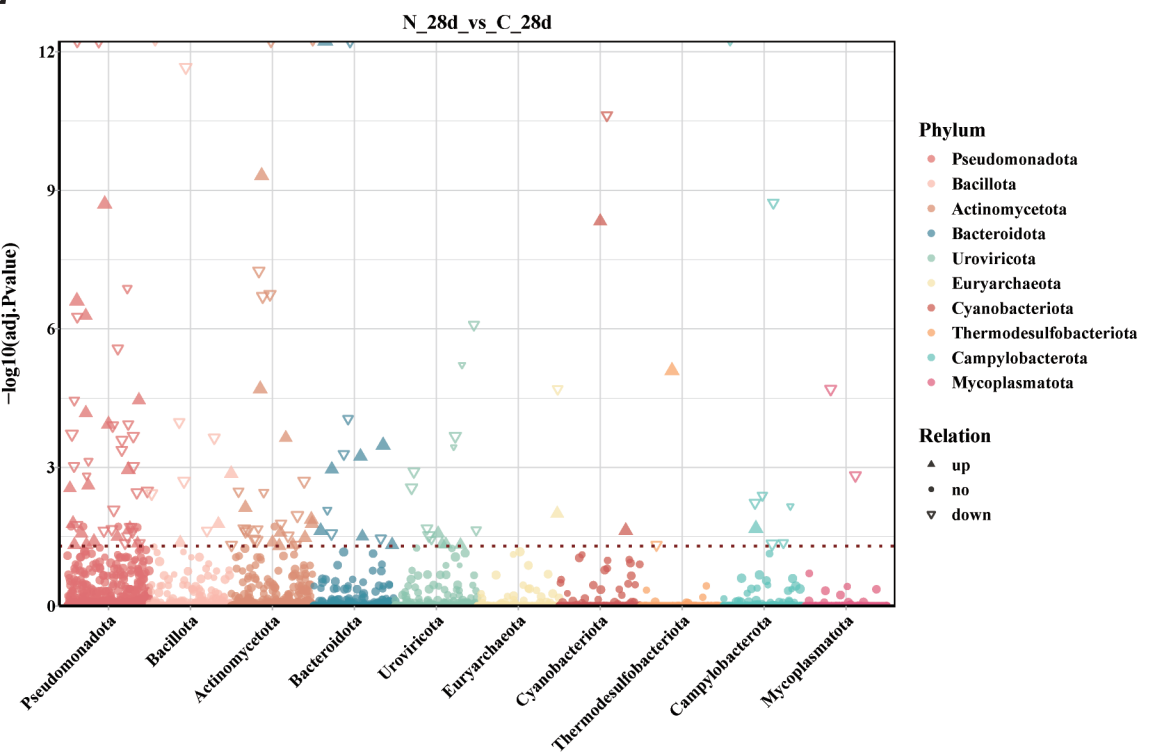

Supplement: Supplementary file 1 [file metabolites-16-00143-s001.zip › Figure S3.pdf]

**A**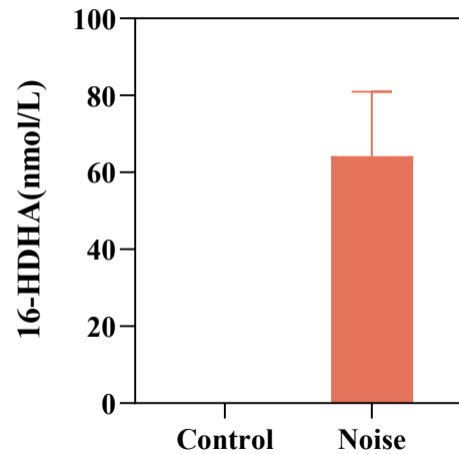**B**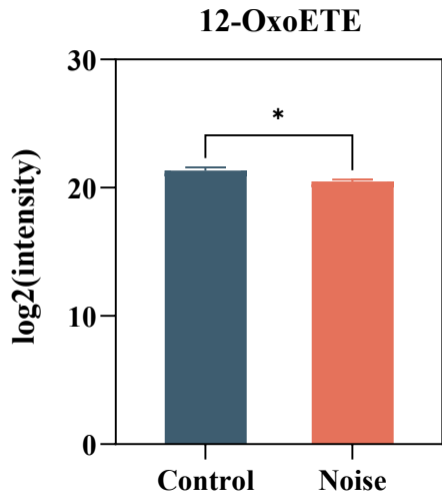**C**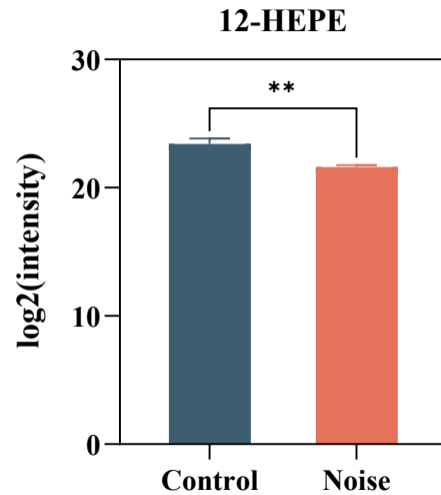**C**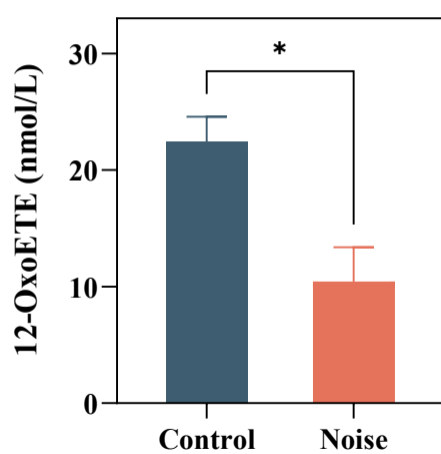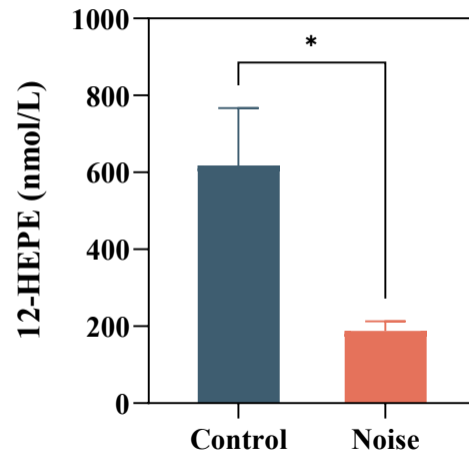

Supplement: Supplementary file 1 [file metabolites-16-00143-s001.zip › Figure S4.pdf]
